# Promoting menstrual health and hygiene—insights from the 2023 World Menstrual Hygiene Day celebration events in the Hohoe municipality in Ghana

**DOI:** 10.3389/fpubh.2024.1406665

**Published:** 2024-09-13

**Authors:** Sitsofe Gbogbo, Israel Wuresah, Phyllis Addo, Senam Klomegah, Emmanuel Gbogbo, Wisdom Axame, Priscilla Klutse, Christopher Makam, Robert Kokou Dowou, Paramount Eli Nelson, Ishmael Boateng, Sarah Odi Mantey, Nuworza Kugbey, Victor Christian Korley Doku, Julie Hennegan, Frank E. Baiden, Lydia Aziato, Fred N. Binka

**Affiliations:** ^1^School of Public Health, University of Health and Allied Sciences, Ho, Ghana; ^2^School of Natural and Environmental Sciences, University of Environment and Sustainable Development, Somanya, Ghana; ^3^Faculty of Life Sciences and Medicine, King’s College London, London, United Kingdom; ^4^Maternal, Child and Adolescent Health Program, Burnet Institute, Melbourne, VIC, Australia

**Keywords:** menstrual health, menstrual rights, World Menstrual Hygiene Day, menstrual health education, cultural acceptance

## Abstract

The 2023 World Menstrual Hygiene Day (WMHD) celebration at the University of Health and Allied Sciences (UHAS) sought to create awareness about menstruation. Toward normalizing menstruation, the 3-day event brought together key stakeholders and engaged community members on various topics relating to menstruation. Among the stakeholders were basic school teachers (13), school children (155), in a 2:1 female vs. male ratio within the ages of 10–17 years, School Health and Education Program Coordinators (2), Health Officers (4), Academicians (15), University students (35), Media representatives (3), and Civil society representatives (130). Particular among these discussions were the biology of menstruation, nutrition related facts during menstruation, sociocultural, and mental health issues surrounding menstruation. These discussions were intended to incite more conversations about menstruation, and contribute toward the agenda 2030 goal of making menstruation a normal fact of life. The paper describes activities conducted to improve menstrual health, reduce period poverty, and involve men in breaking the stigma around menstruation. This contributes to creating supportive environments for menstruating individuals. Meanwhile, the lessons from the activities to celebrate WMHD in Ghana are relevant for other communities to consider replicating with consideration for contextual differences.

## 1 Introduction

### 1.1 Description of the nature of the problem being addressed

Menstruation, a natural biological process in the reproductive system of females, is often accompanied by resource and socio-cultural challenges (1, 2). These challenges contribute to a range of physical, social, and psychological difficulties experienced by menstruating individuals—including girls, women, transgender, and non-binary persons (3).

One significant challenge is the lack of access to menstrual hygiene products—termed as period product insecurity or period poverty, particularly in low- and middle income countries and marginalized communities (4). The unreliable access to sufficient menstrual products or lack of access to products of choice (5, 6) are globally recognized challenge for menstruating individuals. Difficulties affording or obtaining hygienic menstrual products can lead to unhygienic practices, discomfort, and embarrassment (2). The financial burden and limited availability of these products pose additional challenges for menstruating individuals. Inadequate sanitation and water facilities pose another challenge for menstruating individuals, particularly in resource-constrained settings (7). The absence of private toilets, clean water, and proper waste management options can compromise menstrual hygiene, increase the risk of infections, and hinder individuals’ daily activities (8).

The lack of awareness and education about menstruation perpetuates myths, misconceptions, and cultural taboos, further reinforcing the stigma associated with this natural process (2, 9). The absence of sufficient information creates societal silence and misinformation about menstruation, which hinder open discussions, create a sense of isolation, and prevent individuals from seeking support. Further, menstruation is often considered impure or shameful, leading to social exclusion, restrictions on daily activities, and mobility limitations for girls and women (2). The gendered nature of menstruation-related issues perpetuates harmful stereotypes, reinforcing the subordination of women and limiting their autonomy and agency. The stigma, deeply rooted in gender inequality and discrimination, is prevalent in many societies especially in low- and middle income countries (9, 10). Likely occurring more often than is, reported, these challenges and stigmas have significant impacts, enveloped in stress, anxiety, and low self-esteem, on the overall well-being of individuals who menstruate (11).

### 1.2 Rationale for the proposed innovation

Amidst the global efforts to increase awareness, demystify menstruation and normalize conversations about menstruation, we set out to educate residents of Hohoe about menstruation for heightened menstruation awareness, and to provide adolescent girls with skills for the production of reusable pads with the latter aimed at increasing access to affordable and sustainable options for managing menstruation.

This community case study reports on the activities undertaken during the 3-day event in Hohoe Ghana under the 2023 WMHD Celebration theme: “*Making menstruation a normal fact of life by 2030*.” WMHD has become a significant platform for advocacy and education around menstrual health and hygiene (12). Starting in 2013 by the German-based non-profit WASH United (13), WMHD is rooted in raising awareness and breaking the silence surrounding menstruation (14). The 2023 event in Hohoe mirrored this mission with a focus on ending period stigma and poverty through awareness creation, stakeholder engagement and skill-based training.

The activities were purposefully curated in accordance with the theme of WMHD 2023. The community health walk was intended to raise visibility and encourage public discourse on menstruation, while the radio reaches a broad audience, including those without access to formal education or the internet. Also, discussing a range of topics ensures comprehensive education on menstruation, addressing both biological and socio-cultural aspects. We made sure to encourage community ownership of the event and leverage local influence to drive change through stakeholder engagement. While other resources for menstrual hygiene exist in other places, the most prevalent approach in Hohoe is the distribution of disposable materials—which are not sustainable. Training girls to make reusable pads could address menstrual product insecurity and promote sustainability.

## 2 Key programmatic elements

The 2023 WMHD celebration at the University of Health and Allied Sciences (UHAS) in Hohoe Ghana was aimed at addressing the challenges and stigma associated with menstruation. The event brought together basic school teachers (13), school children (155) in a 2:1 female vs. male ratio, within the ages of 10–17 years, School Health and Education Program Coordinators (2), Health Officers (4), Academicians (15), University students (35), Media representatives (3), and Civil society representatives (130).

The line-up of activities consisted of a community health walk, and a radio talk show on May 26, 2023 designed to create awareness, a seminar on Reusable pads (RUPs) on May 27, 2023 targeted at reducing period poverty and climaxing with a symposium on May 28, 2023, where various stakeholders were engaged. The details of the activities are described below.

### 2.1 Community health walk

The walk was intended to raise awareness and incite conversations about menstruation as a means of normalizing menstruation. With over 150 participants consisting of WMHD 2023 committee members and volunteers of various university student organizations, walkers carried banners and placards through the streets of Hohoe. As part of the walk, committee members made stops at markets, shops, schools, and homes near the streets to provide simple understandable facts about menstruation with the goal of increasing knowledge about menstruation and dispelling myths and misconceptions. Given the importance attached to transformational change regarding perceptions about menstruation, the walk targeted a variety of audiences ranging from young adolescents to older adults.

### 2.2 Radio talk

The radio program was designed to reach a wider audience within the frequency reach of the radio stations, potentially reaching over 100,000 listeners across two sessions amounting to 4 h, with the same aim of raising awareness. The talk covered key issues such as the biological aspects of menstruation, the significance of nutrition during menstruation, sociocultural influences on menstruation, and the impact of menstruation on mental health, specifically addressing topics like depression, anxiety, and stress that could result from cultural restrictions. The program was tailored to foster comprehension, confront societal taboos, and diminish the social stigma associated with menstruation. A group of experts with backgrounds in medicine and clinical epidemiology, nutrition and food science, behavioral sciences, clinical psychology and mental health, and education were involved in delivering the talks. The details of the program are presented in Table 1.

**Table 1:** Detailed radio program activities.

	*Topic of discussion*	*What was discussed*
1.1	Biological aspects of menstruation	What happens before the blood flow? What is the reproductive importance of ovulation? What happens after ovulation? What is the importance of the blood flow to the reproductive system of women? Is menstruation abnormal? Biological challenges associated with menstruation – dysmenorrhea, amenorrhea, oligomenorrhea, menorrhea
1.2	Nutrition during menstruation	The impact of nutrition during menstruation Menstruation and balance diet Local food types for improved menstruation
1.3.1	Sociocultural influence of menstruation	Leads to stereotypes Discrimination Restrictions and isolations Stigma
1.3.2	Normalizing menstruation	Make conscious efforts to increase awareness Male education and involvement in menstruation interventions Creating supportive environment for menstruation management – water and sanitation facilities Advocating for tax-free pads
1.4	Menstruation and mental health	The link between menstruation and depression, stress and anxiety Discrimination, stigma and self-worthlessness Seeking help in the face of stigma and self-worthlessness

#### 2.2.1 Biological aspects of menstruation

The discussions highlighted the broader biological significance of menstruation. The talk centered on how menstruation helps to clear out and renew the endometrium, removing damaged or aged cells, tissue, and debris from the uterus (15). For understanding the intricate hormonal regulation and cyclic nature of menstruation, the message was tailored to provide insights into the natural variations and changes that occur within the bodies of females. This could provide knowledge that leads the listeners to embrace and accept menstruation as a normal and essential part of the lives of females.

#### 2.2.2 Nutrition during menstruation

The primary focus of this session was to provide participants with essential information and guidance regarding nutritional support during menstruation. Recognizing the importance of maintaining proper nourishment during this time, the workshop aimed to empower participants with knowledge of food items or nutrients that are readily available in their communities to support their overall well-being.

Menstrual blood loss can result in iron depletion and, potentially, iron-deficiency anemia. To replenish iron levels, participants were informed about the inclusion of iron-rich foods in their diet, such as leafy greens, legumes, nuts, and lean meats (16). Moreover, combining these iron-rich foods with sources of vitamin C, such as citrus fruits or bell peppers, were suggested, as vitamin C enhances iron absorption.

Beyond iron, the significance of other essential nutrients in supporting menstrual health were highlighted. Calcium, which plays a crucial role in maintaining bone health, was discussed, and participants were encouraged to incorporate calcium-rich foods, such as dairy products, fortified plant-based kinds of milk, leafy greens, and sesame seeds (17) into their diets. Additionally, the potential benefits of magnesium for reducing menstrual cramps and promoting relaxation were discussed (18). Listeners were informed about food sources rich in magnesium, including whole grains, nuts, and seeds. The role of omega-3 fatty acids in alleviating menstrual symptoms due to their anti-inflammatory properties was also discussed, with sources like fatty fish, flaxseeds, and walnuts being recommended (19).

#### 2.2.3 Sociocultural influence of menstruation

There are several social and cultural constructions about menstruation with many of them leading to discrimination, stereotypes and stigma. This talk was intended to create awareness among community members, especially traditional leaders and males, to ensure a more inclusive and supportive environment for menstruating individuals. The talk highlighted the need to make conscious efforts in reorientation for increased awareness about menstruation.

#### 2.2.4 Menstruation and mental health

Mental health issues during menstruation, specifically focusing on the mediation of stigma and discrimination, were discussed. This session also focused on drawing the link between various forms of stereotypes and the development of mental health problems. Recommendations on how to manage these challenges and promote mental well-being among menstruating individuals were made. Several strategies were discussed, including the importance of having trained counselors in schools, fostering peer support groups, and seeking assistance from mental health experts.

### 2.3 Symposium

The symposium, segmented into two sessions and lasting 8 h (8 a.m.–5 p.m.), featured a diverse group of participants, including both male (50) and female (105) students, and teachers from over 10 basic schools in Hohoe, community stakeholders (opinion leaders) such as the chiefs, elders, and queen mothers of the traditional area, whose participation were of paramount interest, population and behavioral science experts, reproductive health experts, food and nutrition scientists, and faculty members of the University of Health and Allied Sciences, making about 250 participants This section adds up to the above stated activities in attempting to increase the global efforts of normalizing menstruation by 2030. The design of the symposium was such that it recognized the influence of traditional leaders in improving menstruation-related discussions, hence their inclusion. Traditional community entry procedures were strictly adhered to during the process of inviting traditional council members. This approach acknowledges the authority of community leaders and their essential role in driving positive change.

The symposium featured rigorous presentations and demonstrations across four key areas: the biological mechanisms underlying the menstrual cycle, the role of nutrition, the sociocultural aspects, and the connection of menstruation to mental health. Following each set of presentations, panel discussions were held that included the presenters as well as two expert reproductive health practitioners from UHAS and the Volta Regional Hospital, Hohoe, allowing for in-depth exploration and discussion of the topics covered.

### 2.4 Capacity building training on reusable pads

This session was held on the final day of the celebration starting at 8 a.m. and finishing at 5 p.m. About 200 students aged 10–17 years (two males volunteered) participated in the training.

This activity was designed to contribute to efforts to end menstrual product insecurity. A seminar was organized to train girls on producing reusable pads and pants liners using clean, simple, and easily accessible materials as outlined by the UNICEF (20). This practical capacity-building activity aimed to empower girls (10–17 years) with valuable skills while promoting sustainable menstrual hygiene practices. Prizes were incorporated into the workshop to encourage active participation and motivate the learning process. Our practical sessions to provide capacity building on reusable menstrual products proved feasible and harnesses the potential advantages of reusable products.

## 3 Setting and population

The intervention was conducted in Hohoe, a municipality in the Volta Region of Ghana, which encompasses a blend of urban and rural areas (21). Hohoe serves as a commercial and administrative hub for surrounding villages, with its population engaging primarily in agriculture, small-scale trade, and various service industries (22). The municipality has a relatively well-developed infrastructure, including schools, health facilities, and markets, but still faces challenges typical of developing regions (22).

The population of Hohoe is approximately 167,000, with a nearly equal gender distribution and a significant proportion of young people (21, 22). Women and girls of reproductive age (15–44) constitute a large segment (43.9%) of the 52.1% female population (23). The cultural landscape of Hohoe is diverse, with various ethnic groups coexisting, each with unique traditions and beliefs that can influence perceptions and practices related to menstrual health and hygiene. However, the Ewe ethnicity is dominant.

## 4 Discussion: lessons learned from WMHD 2023 and opportunities for future research

### 4.1 Male involvement in menstrual hygiene management

Men’s growing involvement in menstruation—evident in the activities undertaken, despite prevailing taboos and myths, signifies a progressive shift toward breaking down societal barriers and fostering inclusivity (24). Traditionally, cultural norms have surrounded menstruation with taboos that exclude men from discussions and awareness initiatives (25). However, a notable change is occurring as more men express a willingness to actively engage in conversations around menstruation, challenging existing societal barriers (1). Their open dialogue and participation could contribute to dispelling myths and misconceptions, fostering a more informed and supportive community. Participating in all the activities of WMHD 2023 indicates that men’s involvement extends beyond personal understanding; it could translate into roles as supportive partners, fathers, and family members. Through active participation in menstruation education, men could contribute to creating a positive environment at home and challenging traditional gender norms. This reinforces a shared responsibility in managing menstrual hygiene.

### 4.2 Advancing understanding and use of reusable pads

Development and use of reusable sanitary pads offer several notable advantages. There are significant environmental benefits as these pads are designed to be washed and reused, thereby reducing the amount of waste generated in comparison to disposable pads (26). Training on RUPs were considered in our activities to highlight sustainability and access to affordable materials for managing menstruation. This emphasis on sustainability aligns with global efforts to minimize the ecological impact of consumer products. Economically, reusable pads provide a cost-effective solution, particularly in regions facing challenges in accessing affordable menstrual hygiene products (27, 28). The affordability not only improves personal well-being but also addresses economic disparities by making these products more widely accessible.

Moreover, the focus on natural and breathable materials in reusable pads stands to enhance user comfort, reducing the risk of irritation and discomfort often associated with synthetic materials (29). Additionally, evolving innovations could ensure that reusable pads continue to improve in design, performance, and user satisfaction.

Vis-a-vis the affordability and sustainability of RUPs, the transition from synthetic pads to RUPs could pose challenges. As deeply ingrained beliefs and practices related to menstrual hygiene may lead to reluctance in certain communities (30), cultural resistance can hinder the widespread adoption of these products. Meanwhile, the initial cost associated with acquiring the right materials could be another challenge. While they offer long-term savings, the upfront investment may pose a barrier, especially in low-income communities (31).

Hygiene concerns may also arise as ensuring proper hygiene with reusable pads involves regular washing and maintenance (28). Thus, implementing RUPs on a large scale could be challenging in areas with limited access to water.

Research efforts would be helpful to identify optimum materials and guidelines to ensure both the effectiveness and safety of reusable options. Also, addressing educational gaps regarding proper usage and maintenance is central to maximizing the benefits of reusable options.

### 4.3 Cultural acceptance and user preferences of RUPs

Cultural acceptance and user preferences are key factors that could influence the adoption of RUPs. The diverse cultural landscape surrounding menstruation introduces varying beliefs and taboos that could impact the acceptance of alternative menstrual hygiene products (30). Understanding the intricate tinges of cultural perspectives is critical in this sense. To optimize use, community engagement is essential to gather direct insights from users and address specific cultural barriers. Additionally, the influence of peer dynamics and community leaders could play a substantial role in shaping attitudes toward RUPs. Education initiatives should extend beyond product knowledge to include comprehensive menstrual health education that fosters cultural awareness and dispels myths.

## 5 Conclusion, recommendations, and limitations

### 5.1 Conclusion and recommendations

The WMHD events in Hohoe in 2023 provide an example of a multi-day celebration to raise awareness about menstruation and combat menstrual product insecurity through hands-on training on RUPs. The initiative aimed to break down societal barriers by providing practical skills and knowledge on the use and maintenance of reusable products, fostering a more informed and open community dialogue. The focus on RUPs was not only to align with sustainability goals and reducing non-biodegradable waste but also to address economic challenges related to menstrual hygiene.

Thus, the events served as a platform for community discussions, challenging societal norms and eliminating taboos associated with menstruation, with an overarching commitment to making menstrual hygiene accessible and affordable for all.

However, further evidences that direct policy around male involvement in MHM, and reusable menstrual pads are required. Hence, we recommend that future research focuses on gathering such data to help advocate for more male involvement in menstrual health and hygiene management and the provision of national guidelines on RUPs production and use in Ghana.

### 5.2 Limitations

This community case study has some limitations due to its methodology and scope. First, the study did not employ formal data collection methods, relying instead on the reflections and perspectives of key stakeholders involved in the event. This limits the ability to provide empirical evidence, as the insights and conclusions are primarily reflective.

Also, the design introduces potential bias. Participants, including event organizers, community leaders, and volunteers, may have subjective views influenced by their roles and interests. This could lead to an over or underrepresentation of positive outcomes and vice versa of challenges or negative aspects.

Lastly, the context-specific nature of this report limits its generalizability. The cultural, social, and economic characteristics of Hohoe, as well as the specific activities undertaken during the WMHD event, are unique to this setting. Consequently, the lessons learned and recommendations derived from this study may not be applicable to other regions or communities with different contexts.

To assess the outcomes or impact of these activities, future initiatives could incorporate formal data collection methods such as surveys, interviews, and focus groups to provide empirical evidence and reduce bias.

## Data Availability

The original contributions presented in the study are included in the article/supplementary material, further inquiries can be directed to the corresponding author.
